# Magnetic control of membrane damage in early endosomes using internalized magnetic nanoparticles

**DOI:** 10.1247/csf.24037

**Published:** 2024-12-27

**Authors:** Yuta Yonekawa, Kazuki Oikawa, Boldbaatar Bayarkhuu, Kizuna Kobayashi, Nana Saito, Ibuki Oikawa, Ryohei Yamada, Yu-han Chen, Koichi Oyanagi, Yuji Shibasaki, Satoru Kobayashi, Yoko Shiba

**Affiliations:** 1 Graduate Course in Biological Sciences, Division of Science and Engineering, Graduate School of Arts and Sciences, Iwate University, Morioka, Japan; 2 Graduate Course in Materials Science and Engineering, Division of Science and Engineering, Graduate School of Arts and Sciences, Iwate University, Morioka, Japan; 3 Graduate Course in Chemistry, Division of Science and Engineering, Graduate School of Arts and Sciences, Iwate University, Morioka, Japan; 4 Department of Biochemical Science and Technology, National Chiayi University, Taiwan

**Keywords:** magnetic nanoparticles, endosomes, membrane damage, organelle

## Abstract

Membrane stiffness is essential for cell migration, tumorigenesis, and development; however, the physical properties of intracellular membrane are poorly characterized. In this study, we internalized 20 nm magnetic nanoparticles (MNPs) into MCF7 human breast cancer cells and applied a magnetic field. We investigated whether magnetic field could induce membrane damage of the early endosomes by analyzing the colocalization of MNPs with galectin 3 (Gal3), a cytosolic protein recruited to the lumen of damaged organelles. We first tried to apply magnetic field by electromagnet, and found a direct-current (DC) magnetic field for five minutes increased the colocalization of the MNPs with Gal3, suggesting that the magnetic field damaged the endosomal membrane. We used a neodymium magnet to apply longer and stronger static magnetic fields. The static magnetic field more than 50 mT for five minutes started to damage endosomes, while 100 mT was the most effective. Longer exposure or higher magnetic field strengths did not induce further membrane damage. We confirmed that a Gal3 positive compartment was also positive for the early endosome marker, EEA1, suggesting that the external magnetic field induced membrane damage in the early endosomes. Our results indicate that a static magnetic field can control the membrane damage in early endosomes using internalized MNPs.

## Graphical Abstract

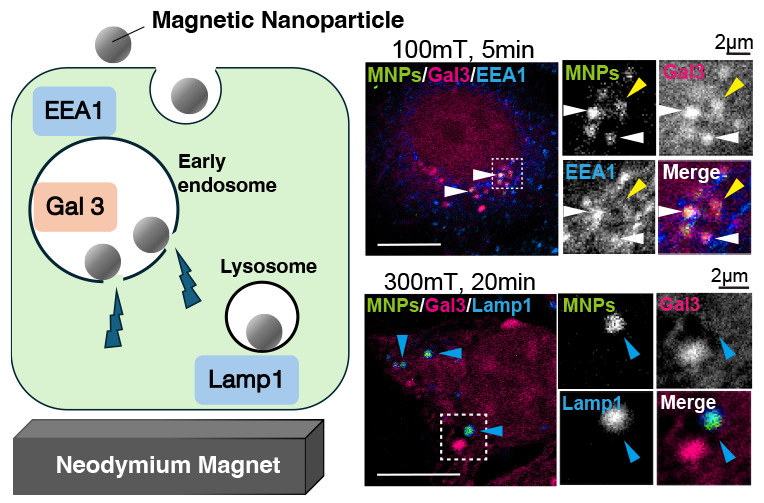

## Introduction

Mammalian cells internalize diverse materials, including nutrients, mineral crystals, and pathogens, through endocytosis. These materials are subsequently trafficked to early endosomes, late endosomes, and lysosomes, where they undergo degradation. The integrity and physical properties of endosomal and lysosomal membranes are vital for cellular survival (Lima *et al.*, 2013). Pathogens such as bacteria and viruses frequently remodel or escape from endosomes and lysosomes to promote their proliferation (Alvarez-Dominguez *et al.*, 1997; Daussy and Wodrich, 2020; Krieger *et al.*, 2014; Liss *et al.*, 2017; Maier *et al.*, 2012; Nguyen *et al.*, 2019; Petrovska *et al.*, 2004; Senerovic *et al.*, 2012). Therefore, the physical properties of organelle membranes are essential to protect cells from their environment.

The amount of force required for luminal cargos to go through the early endosomes’ membrane is not established. The force necessary to rupture a bilayer membrane has been estimated to 60–150 pN, 180–450 mT, when using magnetic nanoparticles (MNPs) with a 10 nm radius (Golovin *et al.*, 2015). MNPs are nanoscale particles typically composed of magnetic elements such as iron. The forces to pull out the plasma membrane by optical tweezers were reported to be 30–60pN (Pontes *et al.*, 2017). In this case, the membrane was pulled out but not ruptured. The force to rupture membrane was reported by atomic force microscopy (AFM) (Penedo *et al.*, 2021). The forces to rupture the plasma and nuclear membranes were approximately 500 pN to rupture the plasma membrane and 1,000 pN for the nuclear membrane, with the radius of the cantilever tip below 20 nm. However, it is not known if this force can be the same in endosomal membrane when luminal cargos penetrate the endosomal membrane, as endosomes are more dynamic and less supported by cytoskeleton than the plasma membrane and the nuclear membrane.

Magnetic hyperthermia is a technique by which an external magnetic field kills cancer cells by heat generated by MNPs internalized into cancer cells (Cazares-Cortes *et al.*, 2019). In this case, an alternating magnetic field (AMF) of high frequency (200–700 kHz) was used. However, cell death can be caused by low-frequency magnetic fields without heating; therefore, the mechanical effects of MNPs have been discussed (Golovin *et al.*, 2015). A recent report described that very little force could disrupt the lysosomes (Lopez *et al.*, 2022). Lopez *et al.* synthesized 40 nm MNPs with a 6 nm iron oxide core with the functionalization of a ligand that binds to its receptor on the plasma membrane. MNPs were internalized for 24 hrs for transport to the lysosome, and cell death was observed under a rotating magnetic field of 40 mT for 2 hrs. They calculated 3 pN of the force was generated from 3,000 MNPs. This is well below the force to be required for rupturing bilayer membrane.

The internalization of mineral crystals or latex beads coated with cationic lipids alone damaged endosomes or phagosomes without additional forces (Chen *et al.*, 2014; Fujita *et al.*, 2013; Maejima *et al.*, 2013; Takahashi *et al.*, 2017). MNPs coated with cationic charges or other ligands that bind to some receptors in the membrane can damage membrane by restricting the motion of proteins or lipids in the membrane by alternating magnetic field (AMF) or round magnetic field (RMF) (Golovin *et al.*, 2015). In this case, no force or very small force is required for membrane damage. However, in terms of controlling the timing of membrane rupture by applying magnetic field, it is beneficial to avoid automatically damage the membrane by cationic charges or ligands to bind to membrane receptors. MNPs with a coating of anionic or no charge without any ligands to bind to the membrane receptors can also be internalized (Calero *et al.*, 2014, 2015). MNPs of 10–50 nm were delivered to the lysosomes for magnetic isolation of intact lysosomes (Le *et al.*, 2022; Tharkeshwar *et al.*, 2017). However, it is not known if MNPs without cationic charges or modification of ligands to bind to membrane receptors can damage the endosomal membrane by applying magnetic field, rather than elongating membrane tubules.

Previously, we prepared 15 nm MNPs of Fe_3_O_4_ (Kawamura *et al.*, 2024). Fe_3_O_4_ MNPs with a diameter smaller than 25 nm exhibit superparamagnetic properties, whereas those larger than 25 nm are ferromagnetic (Krishnan, 2010). Superparamagnetic nanoparticles have no magnetization in the absence of an external magnetic field but become magnetized when exposed to it. Hydrophobic MNPs dispersed in water tend to chemically self-aggregate, which can induce ferromagnetism and exacerbate aggregation. To prevent such aggregation, we coated 15 nm MNPs with hyper-branched polyglycidol (hbPGYLD-MNPs), enhancing their water solubility. The MNPs were subsequently labeled with fluorescein isothiocyanate (FITC) for detection via confocal microscopy, resulting in 20 nm MNPs. Importantly, the MNPs were not modified with cationic charges or ligands targeting membrane molecules.

In this study, we determined whether our MNPs without cationic charges or specific ligands that bind to membrane receptors can damage the early endosomal membrane by applying magnetic field. We evaluated the membrane damage mainly by colocalization of MNPs with Galectin-3 (Gal3), that binds to the luminal glycans of organelles. When pathogens or non-organic materials damage the organelles, Gal3 moves from the cytosol to the lumen of the organelles (Maier *et al.*, 2012; Paz *et al.*, 2010; Thurston *et al.*, 2012). Therefore, Gal3 is often used as a marker of membrane damage. We also determined the magnetic field intensity required to damage endosomal membranes by internalized MNPs. We found MNPs can colocalize with Gal3 by applying static magnetic field, and the early endosomes can be damaged more than 20–50 mT of static magnetic field for 5 min, and maximum to be 100 mT. Our results suggested that magnetic field can control membrane damage by internalized MNPs without specific ligands or cationic charges, and the magnetic field required for membrane damage is lower than that for hyperthermia.

## Results

### MNP internalization into MCF7 cells

**Fig. 1:**
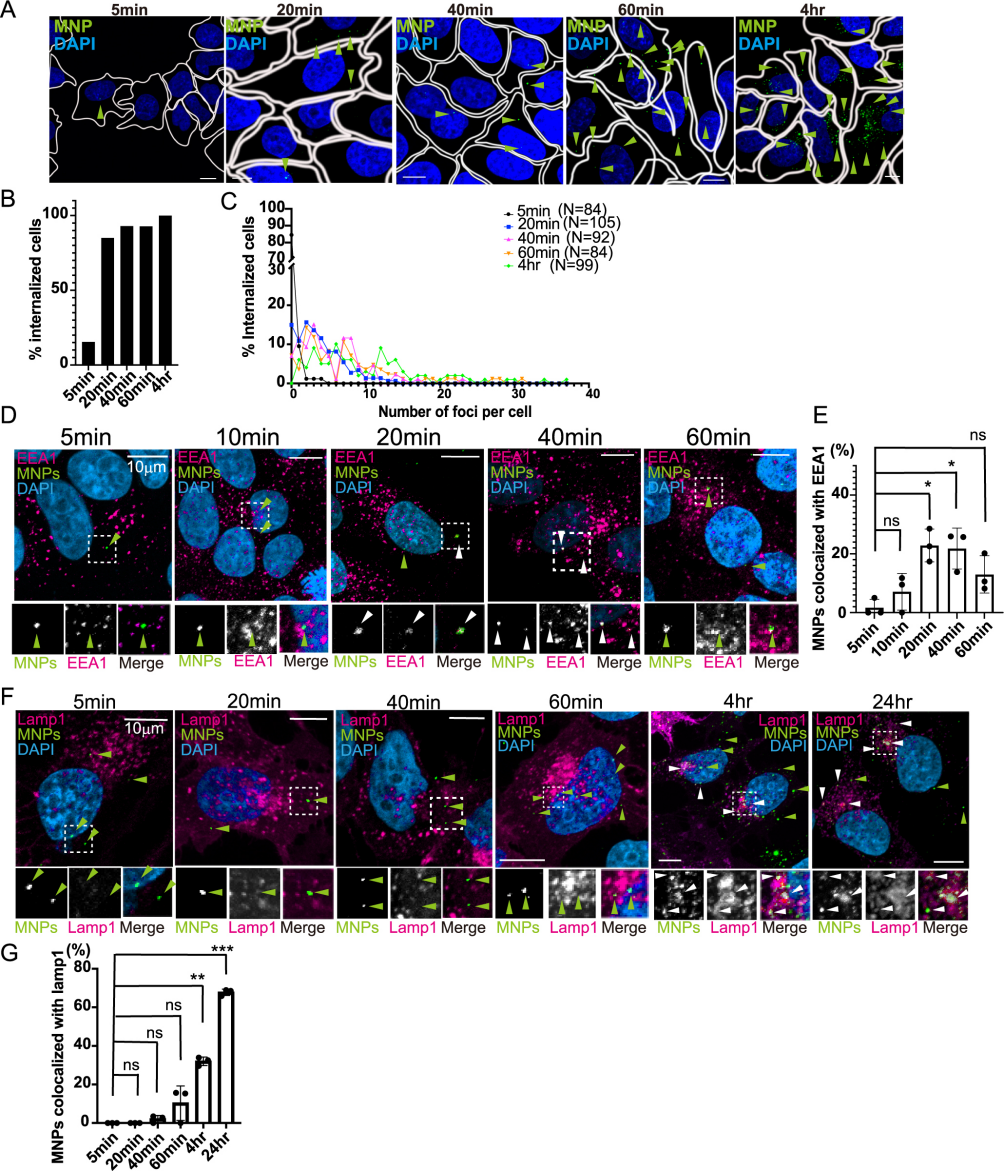
Internalization of MNPs (A) MCF7 cells were incubated with 100 μg/ml MNPs (green) for 5, 20, 40, 60 min, and 4 hrs. After fixation and DAPI staining (blue) to label the nuclei, images were acquired using confocal microscopy. The cell outlines are indicated by white lines, and green arrowheads denote MNPs internalized within MCF7 cells. Scale bar; 10 μm. Internalized MNPs were observed via confocal microscopy, with their numbers increasing over time. (B) For the experiments in (A), the percentage of cells internalizing MNPs was calculated. The total number of cells analyzed is indicated in (C). (C) For the experiments in (A), the number of MNP foci per cell was counted, and the distribution of cells with different numbers of foci was calculated as a percentage relative to the total cell count. More than 28 cells per experiment were analyzed, and the experiments were repeated three times. (D) MNPs (green) were internalized into MCF7 cells for the indicated durations. Cells were fixed and stained with anti-EEA1 antibody (magenta) and DAPI (blue). Green arrowheads indicate MNPs that do not colocalize with EEA1, while white arrowheads indicate MNPs that colocalize with EEA1. Dashed-line regions are enlarged in the insets. Scale bar: 10 μm. (E) From the experiments in (D), the ratio of MNPs colocalized with EEA1 to the total number of internalized MNPs was calculated. More than 10 cells were analyzed per experiment, and the experiment was repeated three times. The bar graphs are presented as mean ± SD. The Kruskal–Wallis test was performed to compare each value with that of 5 min. *p<0.05; ns, not significant. (F) MCF7 cells overexpressing hLAMP1-mScarletI (magenta) were incubated with MNPs (green) for the indicated durations, fixed, and stained with DAPI (blue). Green arrowheads indicate MNPs that do not colocalize with Lamp1, while white arrowheads indicate MNPs that colocalize with Lamp1. Dashed-line regions are enlarged in the insets. Scale bar: 10 μm. (G) From the experiments in (F), the ratio of MNPs colocalized with Lamp1 to the total number of internalized MNPs was calculated as described in (E). More than 10 cells were analyzed per experiment, and the experiment was repeated three times. The bar graphs are presented as mean ± SD. The one-sample t-test and Wilcoxon test were performed to compare each value with that of 5 min. **p<0.01; ***p<0.001; ns, not significant. Colocalization was first observed after 40 min and gradually increased over time.

To determine the optimal condition for delivering our MNPs (hbPGYLD-Fe_3_O_4_-FITC) to the early endosomes, we first assessed the internalization rate of MNPs. MCF7 human breast cancer cells were incubated with 100–200 μg/ml of MNPs for 5, 20, 40, and 60 min, as well as 4 hrs. Cells were fixed and stained with DAPI to label the nuclei, and images were captured using confocal microscopy. Although the foci of MNPs were very small, their signals were detectable within cells (Fig. 1A). The percentage of cells internalizing MNPs relative to the total cell population was quantified (Fig. 1B). After 20 min, 85% of cells had internalized MNPs, and this percentage increased to over 90% after 40 min (Fig. 1B). We observed each cells internalize different number of MNPs, therefore we also calculated the percentage of cells that have each number of foci (Fig. 1C). We observed maximum 24 MNPs foci was found in 40 min of internalization, while in 4 hrs, 37 MNPs foci were found (Fig. 1C), indicating that a longer incubation time is correlated with a larger number of foci per cell.

Next, we internalized MNPs for 5, 10, 20, 40 and 60 min and stained with the early endosome marker, EEA1, to determine the time required for MNPs to reach the early endosomes (Fig. 1D). We found 20–40 min incubation resulted in the highest colocalization with EEA1 (Fig. 1E, 5 min; 0%, 10 min; 7.1%, 20 min; 22.8%, *p*<0.05, 40 min; 21.8%, *p*<0.01, 60 min; 13.0%). In 60 min, we observed less colocalization of MNPs with EEA1. We assume that MNPs passed through the early endosomes and were transported to the lysosomes. We determined when the MNPs reached the lysosomes by analyzing their colocalization with the lysosome marker Lamp1 (Fig. 1F). We expressed hLAMP1-mScarletI in MCF7 cells and incubated MNPs for 5, 20, 40, 60 min, 4 hrs, and 24 hrs. After 40 min, only 2.1% of the MNPs were colocalized with Lamp1. This colocalization increased to 10.3% in 60 min, 32.0% in 4 hrs, and 67.8% in 24 hrs (Fig. 1G). The results indicate that MNPs reach the early endosomes in 20–40 min and lysosomes after 60 min and accumulate in the lysosomes even after 24 hrs.

### Gal3 recruitment in LLOMe-treat cells

To evaluate whether internalized MNPs can damage the membrane under a magnetic field, we examined Galectin 3 (Gal3), that is recruited to MNPs. To validate our detection of Gal3 recruitment, we overexpressed mCherry-Gal3 in MCF7 cells and treated them with L-leucyl-L-leucine methyl ester (LLOMe), a compound known to damage lysosomes (Jia *et al.*, 2020; Repnik *et al.*, 2017). We analyzed colocalization between mCherry-Gal3 and EEA1 and Lamp1 in LLOMe-treated cells (Supplementary Fig. 1A and B). As expected, LLOMe-treated cells exhibited Gal3 foci as reported, with significant colocalization between Gal3 and Lamp1 (Supplementary Fig. 1B and D, DMSO; 26.3%, LLOMe; 60.2%, *p*<0.01). Conversely, no significant colocalization of Gal3 with EEA1 was observed (Supplementary Fig. 1A and C, DMSO; 7.1%, LLOMe; 5.7%, not significant). These findings confirmed that LLOMe selectively damages lysosomes while sparing early endosomes, and membrane damage can be detected through the colocalization of Gal3 with EEA1 or Lamp1.

### Magnetic field application using an electromagnet

**Fig. 2:**
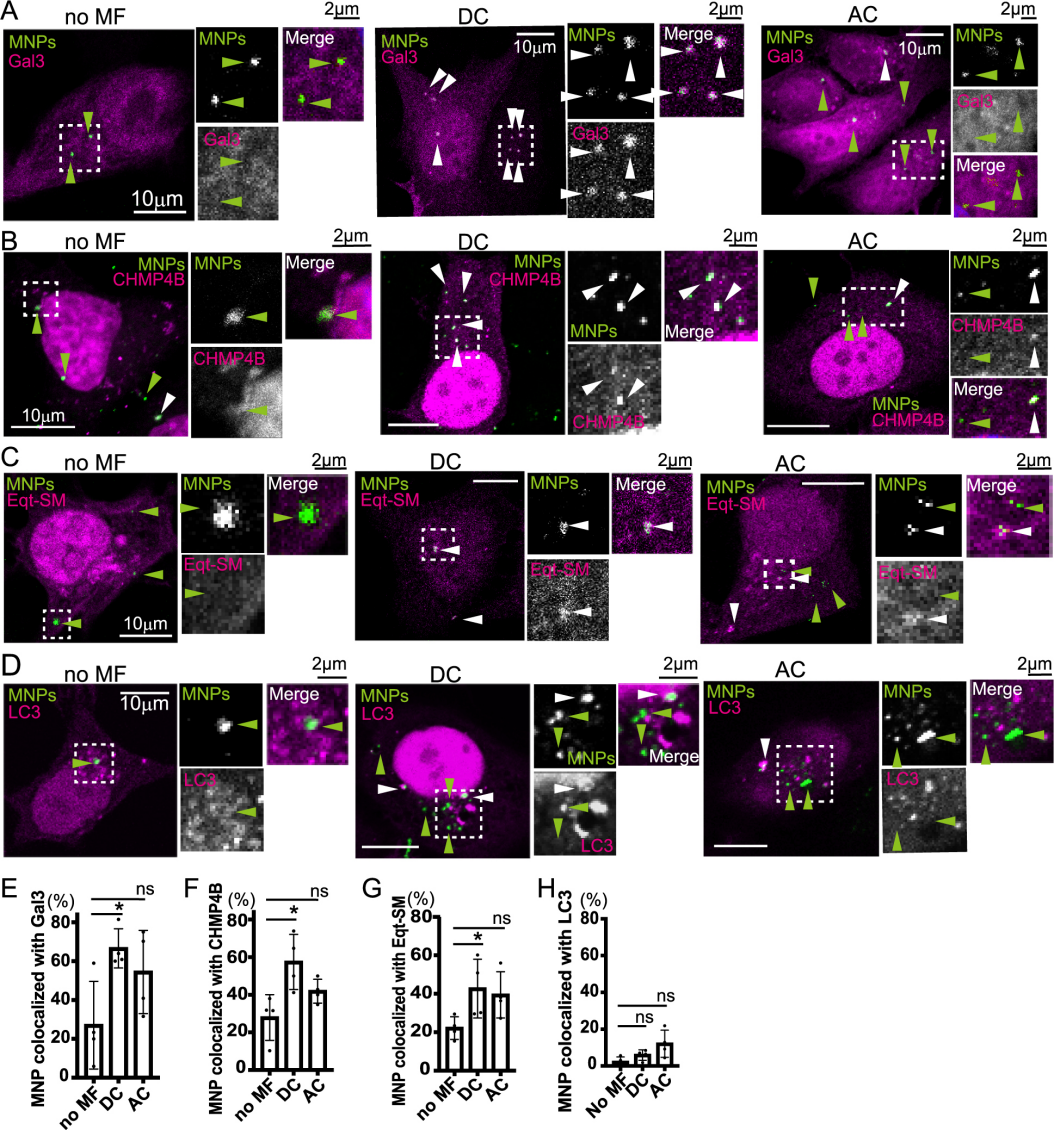
The DC magnetic field induces membrane damage (A) MCF7 cells transfected with mCherry-Gal3 (magenta) were incubated with MNPs (green) for 20 min to allow internalization. After washing, a DC or AC magnetic field of 20 mT was applied using an electromagnet for 5 min. No magnetic field (no MF) was used as a control. Cells were fixed and stained with DAPI (not shown). Green arrowheads indicate MNPs that do not colocalize with Gal3, while white arrowheads indicate MNPs colocalized with Gal3. Scale bar; 10 μm. The dashed-line regions are enlarged in the insets, with their respective scale bars at 2 μm. Application of a DC magnetic field increased the colocalization of MNPs with Gal3. (B) MCF7 cells transfected with CHMP4B-DsRed (magenta) were treated with MNPs (green) and processed as described in (A). Green arrowheads indicate MNPs, and white arrowheads indicate MNPs colocalized with CHMP4B. (C) MCF7 cells transfected with Eqt-SM-DsRed (magenta) were treated with MNPs (green) and processed as described in (A). Green arrowheads indicate MNPs, and white arrowheads indicate MNPs colocalized with Eqt-SM. (D) MCF7 cells transfected with DsRed-LC3 (magenta) were treated with MNPs (green) and processed as described in (A). Green arrowheads indicate MNPs, and white arrowheads indicate MNPs colocalized with LC3. (E) The percentage of MNPs colocalized with Gal3 relative to the total internalized MNPs from (A) was calculated. More than 10 cells were analyzed per experiment, and the experiment was repeated four times. The bar graphs are presented as mean ± SD. The Kruskal–Wallis test was performed to compare each value with that of no MF. *p<0.05; ns, not significant. Applying a DC magnetic field increased the colocalization of MNPs with Gal3. (F) The data from (B) were quantified as described in (E). (G) The data from (C) were quantified as described in (E). (H) The data from (D) were quantified as described in (E).

To determine whether application of magnetic field damaged the early endosomes by MNPs, we internalized MNPs for 20 min to deliver MNPs to the early endosomes, washed excess MNPs from the cell surface, and applied a magnetic field. An electromagnet was employed to compare the effect of magnetic fields of a direct current (DC) or alternating current (AC) on the endosome membranes. The electromagnet was positioned at the bottom of a 24-well plate (Supplementary Fig. 2A) and connected to a power supply. A magnetic field with a maximum intensity of 20 mT was generated using the electromagnet driven by either 2 V DC or 6 V AC at 0.5 Hz (Supplementary Fig. 2B). The magnetic field was applied for 5 min under these conditions. Cells were fixed and visualized using confocal microscopy. Colocalization analysis revealed that MNPs with overexpressed mCherry-Gal3 exhibited increased colocalization under DC magnetic field compared to no magnetic field (no MF) (Fig. 2A, white arrowheads). The increase in colocalization induced by DC magnetic field, but not AC magnetic field, was statistically significant (Fig. 2E; no MF, 27.0%; DC, 66.6%, *p*<0.05; AC, 54.4%, ns).

To confirm the results by Gal3, we investigated additional molecules associated with damaged organelles. Specifically, we analyzed the colocalization of MNPs with CHMP4B, a component of the ESCRT-III complex, which is recruited to damaged endosomes and lysosomes to facilitate membrane repair (Mercier *et al.*, 2020; Radulovic *et al.*, 2018; Skowyra *et al.*, 2018; Vietri *et al.*, 2020). CHMP4B-DsRed was overexpressed in MCF7 cells, and colocalization with MNPs was assessed. Under a DC magnetic field, MNPs displayed significant colocalization with CHMP4B, whereas no significant colocalization was observed under an AC magnetic field (Fig. 2B, white arrowheads, Fig. 2F; no MF, 27.9%; DC, 57.4%, *p*<0.05; AC, 41.9%, ns).

Next, we investigated the colocalization of MNPs with Eqt-SM, an engineered variant of Equinatoxin II designed to detect sphingomyelin exposed to the cytosol upon the lysosomal or phagosomal damage (Boyle *et al.*, 2023; Deng *et al.*, 2016; Niekamp *et al.*, 2022). Under a DC magnetic field, we observed an increase in the colocalization of the MNPs with Eqt-SM (Fig. 2C, white arrowheads, Fig. 2G; no MF, 22.2%; DC, 42.7%, *p*<0.05; AC, 39.4%, ns). However, the increase was less pronounced compared to that observed with Gal3 or CHMP4B.

Finally, we analyzed the colocalization of MNPs with LC3, an autophagy marker associated with degradation of damaged lysosomes or phagosomes (Dupont *et al.*, 2009; Fujita *et al.*, 2013; Hung *et al.*, 2013; Maejima *et al.*, 2013; Thurston *et al.*, 2012). No significant increase in MNP colocalization with LC3 was detected under DC or AC magnetic fields (Fig. 2D and H; no MF, 2.2%; DC, 5.9%, ns; AC, 12.1%, ns). Autophagy is a process involving the degradation of damaged organelles; however, we did not observe an increase of LC3 recruitment in this condition.

### Static magnetic field application with a neodymium magnet

**Fig. 3:**
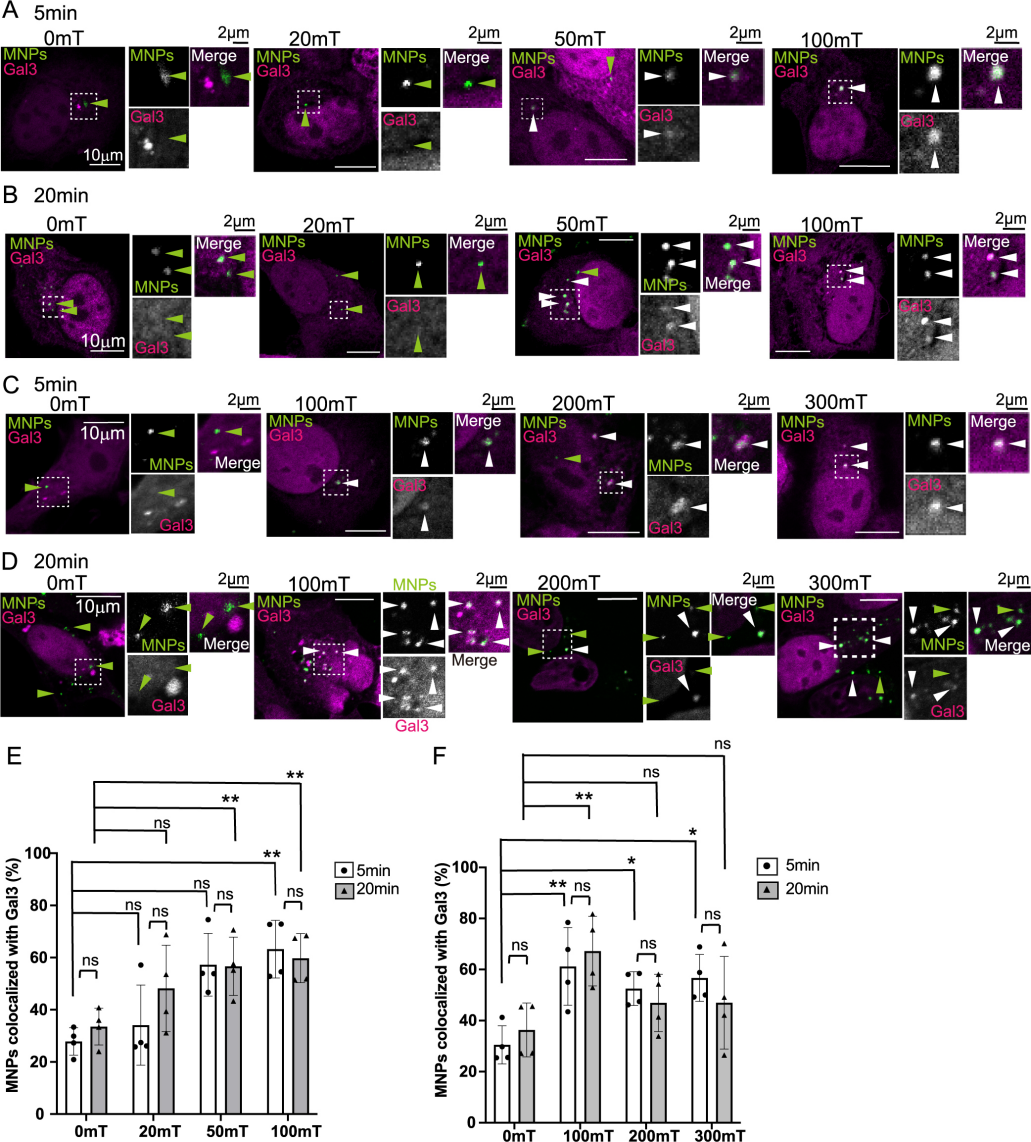
A larger DC magnetic field increases the colocalization between MNPs and Gal3 (A) MCF7 cells were transfected with mCherry-Gal3 (magenta) and incubated with MNPs (green) for 20 min to allow internalization. The cells were washed, and a magnetic field was applied as indicated using a neodymium magnet for 5 min. Cells were then fixed and stained with DAPI (not shown). Green arrowheads indicate MNPs internalized in the cells, while white arrowheads indicate MNPs colocalized with Gal3. Scale bar; 10 μm. The dashed-line regions are enlarged in the insets, with their respective scale bars at 2 μm. (B) MCF7 cells were processed as described in (A), and a magnetic field was applied as indicated for 20 min. (C) MCF7 cells were processed as described in (A), and a magnetic field was applied as indicated for 5 min. (D) MCF7 cells were processed as described in (A), and a magnetic field was applied as indicated for 20 min. (E) The percentage of MNPs colocalized with Gal3 relative to the total internalized MNPs was calculated from experiments (A) and (B). The experiment was repeated four times, and the mean ± SD is presented by the bar graph. White bars represent data for 5 min applications, while grey bars represent data for 20 min applications. A two-way ANOVA with Sidak’s multiple comparison test was used to analyze magnetic field intensity and application duration. *p<0.05; **p<0.01; ns, not significant. (F) The percentage of MNPs colocalized with Gal3 relative to the total internalized MNPs was calculated from experiments (C) and (D) as described in (E). A two-way ANOVA with Sidak’s multiple comparison test was performed as described in (E). *p<0.05; **p<0.01; ns, not significant. Note that colocalization between MNPs and Gal3 was not significantly different at 200 or 300 mT for 20 min.

In our experiments, DC magnetic field was more efficient than AC in inducing endosomal membrane damage using an electromagnet. Therefore, we utilized a neodymium magnet to apply DC-induced magnetic fields at various intensities and durations. Neodymium magnets are recognized for their strength and cost-effectiveness. We adjusted the distance between the cells and the neodymium magnet to apply different magnetic field intensities (Supplementary Fig. 2C). This setup enabled the application of magnetic field in CO_2_ incubator (Supplementary Fig. 2D), ensuring better cell viability during extended magnetic field exposure compared to the electromagnet setup. To evaluate membrane damage, we focused on colocalization of Gal3 with MNPs as Gal3 was more sensitive than other markers for detecting changes in localization (Fig. 2). We overexpressed mCherry-Gal3 and internalized the MNPs for 20 min. The cells were subsequently washed and incubated on the neodymium magnet at 20, 50, and 100 mT for 5 min at 37°C. Higher magnetic field strengths increased the colocalization of MNPs with Gal3 (Fig. 3A). At 100 mT, colocalization significantly increased compared to 0 mT (Fig. 3E; 5 min, 0 mT: 27.8%; 20 mT: 34.1%; 50 mT: 57.2%; 100 mT: 63.2%, *p*<0.01).

These results suggest that magnetic fields exceeding 100 mT applied for 5 min effectively damage the endosomal membrane. Prolonged exposure (20 min) with the same magnetic field produced similar results, with stronger fields enhancing MNP-Gal3 colocalization (Fig. 3E). Colocalization increased with 50 and 100 mT fields (Fig. 3E; 20 min, 0 mT: 33.5%; 20 mT: 48.2%; 50 mT: 56.7%, *p*<0.01; 100 mT: 59.8%, *p*<0.01). However, no significant difference was observed between 5- and 20-min durations (Fig. 3E). These findings suggest that magnetic fields exceeding 50 or 100 mT recruit Gal3 and that prolonged application does not exacerbate endosomal damage.

We further investigated the effects of stronger magnetic fields, 100, 200, and 300 mT for 5 min (Fig. 3C). Colocalization between the MNPs and Gal3 increased at 100, 200 and 300 mT compared with 0 mT (Fig. 3F; 5 min, 0 mT: 30.5%; 100 mT: 61.2%, *p*<0.01; 200 mT: 52.0%, *p*<0.05; 300 mT: 56.7%, *p*<0.05). To evaluate whether prolonged magnetic field application enhances endosomal membrane damage, we applied magnetic fields of 100, 200, and 300 mT for 20 min (Fig. 3D). While colocalization of MNPs with Gal3 increased significantly at 100 mT, no significant differences were observed at 200 and 300 mT compared to 0 mT (Fig. 3F; 20 min, 0 mT: 36.3%; 100 mT: 67.2%, *p*<0.01; 200 mT: 46.9%, ns; 300 mT: 47.0%, ns). Furthermore, comparisons between 5- and 20-min applications showed no significant differences (Fig. 3F). These results raise the possibility that MNPs might have damaged the endosomal membrane within 5 min, and subsequently exited the early endosomes after a 20 min application of a 200- or 300-mT magnetic field.

### Magnetic field with MNPs damaged the early endosomal membrane

**Fig. 4:**
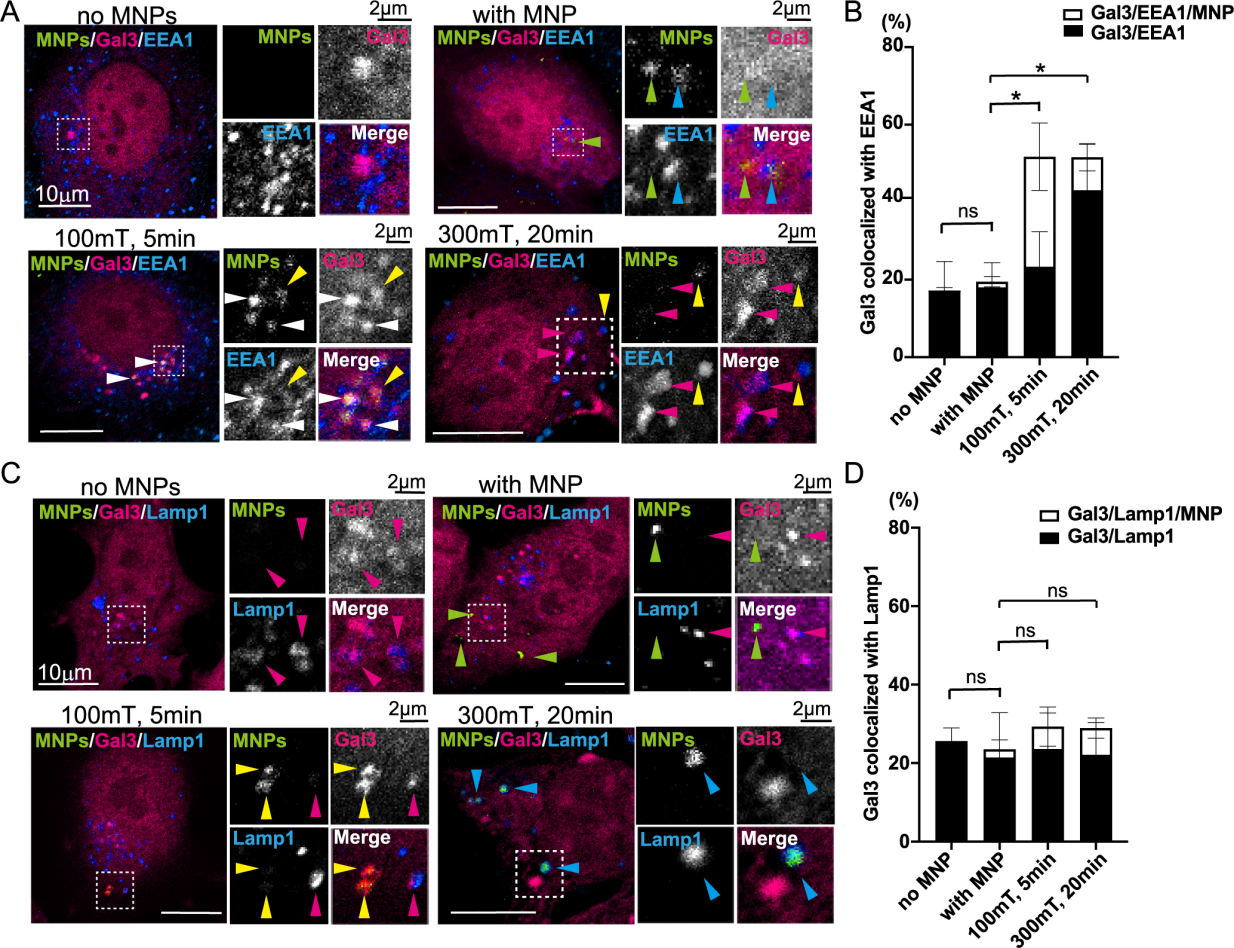
Application of magnetic field damages the membrane of early endosomes (A) MCF7 cells transfected with mCherry-Gal3 (magenta) were incubated with MNPs (green) for 40 min to allow internalization. After washing, a magnetic field was applied as indicated. The cells were fixed, permeabilized with digitonin, and stained for EEA1 (blue). Scale bar; 10 μm. The dashed-line regions are enlarged in the insets, with their respective scale bars at 2 μm. Green arrowheads indicate MNPs without colocalization; blue arrowheads indicate MNPs colocalized with EEA1; yellow arrowheads indicate MNPs colocalized with Gal3; magenta arrowheads indicate Gal3 foci colocalized with EEA1 but without MNPs; white arrowheads indicate Gal3 foci colocalized with both EEA1 and MNPs. Note that triply colocalized Gal3 foci were observed under 100 mT for 5 min. (B) The ratio of Gal3 foci colocalized with EEA1 to the total Gal3 foci was calculated from the experiment described in (A). More than 10 cells were analyzed, and the experiment was repeated four times. Black bars represent the mean of the ratio of Gal3 foci colocalized with EEA1 but not with MNPs. White bars represent the mean of Gal3 foci colocalized with both EEA1 and MNPs. Mann–Whitney test results for total Gal3 foci colocalized with EEA1 (black plus white bars) are shown. *p<0.05; ns, not significant. The values for the white bars and the statistical analysis results are described in the text. (C) MCF7 cells transfected with mCherry-Gal3 (magenta) were processed as described in (A) and stained for Lamp1 (blue). Dashed-line regions are enlarged in the insets. Magenta arrowheads indicate Gal3 foci colocalized with Lamp1 but without MNPs; green arrowheads indicate MNPs without colocalization; yellow arrowheads indicate MNPs colocalized with Gal3; blue arrowheads indicate MNPs colocalized with Lamp1. Note that MNPs were colocalized either with Gal3 or Lamp1. (D) The ratio of Gal3 foci colocalized with Lamp1 to the total Gal3 foci was calculated from the experiment described in (C). More than 10 cells were analyzed, and the experiment was repeated four times. Black bars represent the ratio of Gal3 foci colocalized with Lamp1 but not with MNPs. White bars represent Gal3 foci colocalized with both Lamp1 and MNPs. Mann–Whitney test results for total Gal3 foci colocalized with Lamp1 (black plus white bars) are shown. ns, not significant. The values for the white bars and the statistical analysis results are described in the text.

To confirm that MNPs can damage early endosomes under an external magnetic field, we analyzed the localization of mCherry-Gal3, MNPs and endogenous EEA1. Permeabilization significantly reduced MNP signal intensity. Consequently, MCF7 cells overexpressing mCherry-Gal3 were incubated with 400 μg/ml MNPs for 40 min to enhance signal detection. Magnetic field conditions of 100 mT for 5 min and 300 mT for 20 min were selected. While Gal3 colocalization with MNPs increased at 100 mT for 5 min, it decreased at 300 mT for 20 min, but not at 100 mT for 20 min (Fig. 3E and F). This observation suggests MNP-Gal3 colocalization was reduced under intense magnetic fields during prolonged exposure. We included the cells without MNPs as a control because overexpression of mCherry-Gal3 occasionally produced foci in the absence of MNPs. Application of a 100 mT magnetic field for 5 min significantly increased the colocalization of Gal3 foci with EEA1 (Fig. 4A), and these foci frequently included MNPs. The ratio of Gal3 foci colocalized with EEA1 to the total Gal3 foci was quantified (Fig. 4B; no MNPs: 17.2%; with MNPs: 19.5%; 100 mT, 5 min: 51.7%, *p*<0.05; 300 mT, 20 min: 51.6%, *p*<0.05). The results indicate the early endosomes were damaged both in 100 mT for 5 min and in 300 mT for 20 min. We further analyzed whether Gal3 and EEA1 foci included MNPs. The ratio of triply colocalized foci (Gal3/EEA1/MNP) was quantified (Fig. 4B, Gal3/EEA1/MNP, white bar; no MNP: 0.4%; with MNP: 1.5%; 100 mT, 5 min: 28.4%; 300 mT, 20 min: 8.5%). A Mann–Whitney test comparing the ratios of Gal3/EEA1/MNP foci between 100 mT for 5 min and 300 mT for 20 min revealed a statistically significant difference (*p*<0.05). These findings suggest that MNPs effectively damage early endosomes under 100 mT for 5 min, as well as under 300 mT for 20 min. However, at 300 mT for 20 min, the localization of MNPs within Gal3/EEA1-positive compartments decreases. These observations are consistent with the hypothesis that MNPs damaged the early endosomes within 5 min and subsequently exited the early endosomes after a 20-min application of a 300 mT magnetic field.

A low magnetic field alone has been reported to induce mitophagy (Toda *et al.*, 2022); therefore, we investigated whether our magnetic field conditions could damage the endosomal membrane. A 300 mT magnetic field was applied for 20 min to MCF7 cells without MNPs. Gal3 colocalization with EEA1 was analyzed in the absence of MNPs and compared to the results shown in Fig. 4B. No significant increase in colocalization of Gal3 with EEA1 was observed following magnetic field application alone (Supplementary Fig. 3B; no MNPs, no MF: 10.3%; no MNP with 300 mT: 20 min, 7.8%; with MNP with 300 mT, 20 min: 51.6%, *p*<0.05). These results suggest that the magnetic field alone does not cause endosomal membrane damage.

To evaluate whether the magnetic field also damages lysosomes, we examined the localization of mCherry-Gal3, MNPs, and endogenous Lamp1 (Fig. 4C). Some Gal3 colocalization with Lamp1 was observed without MNPs; however, this colocalization did not increase in the presence of MNPs and under magnetic field conditions of 100 mT for 5 min and 300 mT for 20 min (Fig. 4D, no MNPs: 25.6%; with MNPs: 23.5%; 100 mT, 5 min: 29.3%; 300 mT, 20 min: 28.9%). We also quantified the ratio of Gal3/Lamp1/MNP foci to total Gal3 foci colocalized with Lamp1 (Fig. 4D, white bar, no MNP: 0%, with MNP: 2.1%, 100 mT, 5 min: 5.7%, 300 mT, 20 min: 6.8%). A Mann–Whitney test comparing the ratios of Gal3/EEA1/MNP between 100 mT for 5 min and 300 mT for 20 min revealed no statistically significant difference. These results suggest that lysosomes were not damaged by MNPs and the magnetic field under these conditions.

### CHMP4B and LC3 recruitment to MNPs

**Fig. 5:**
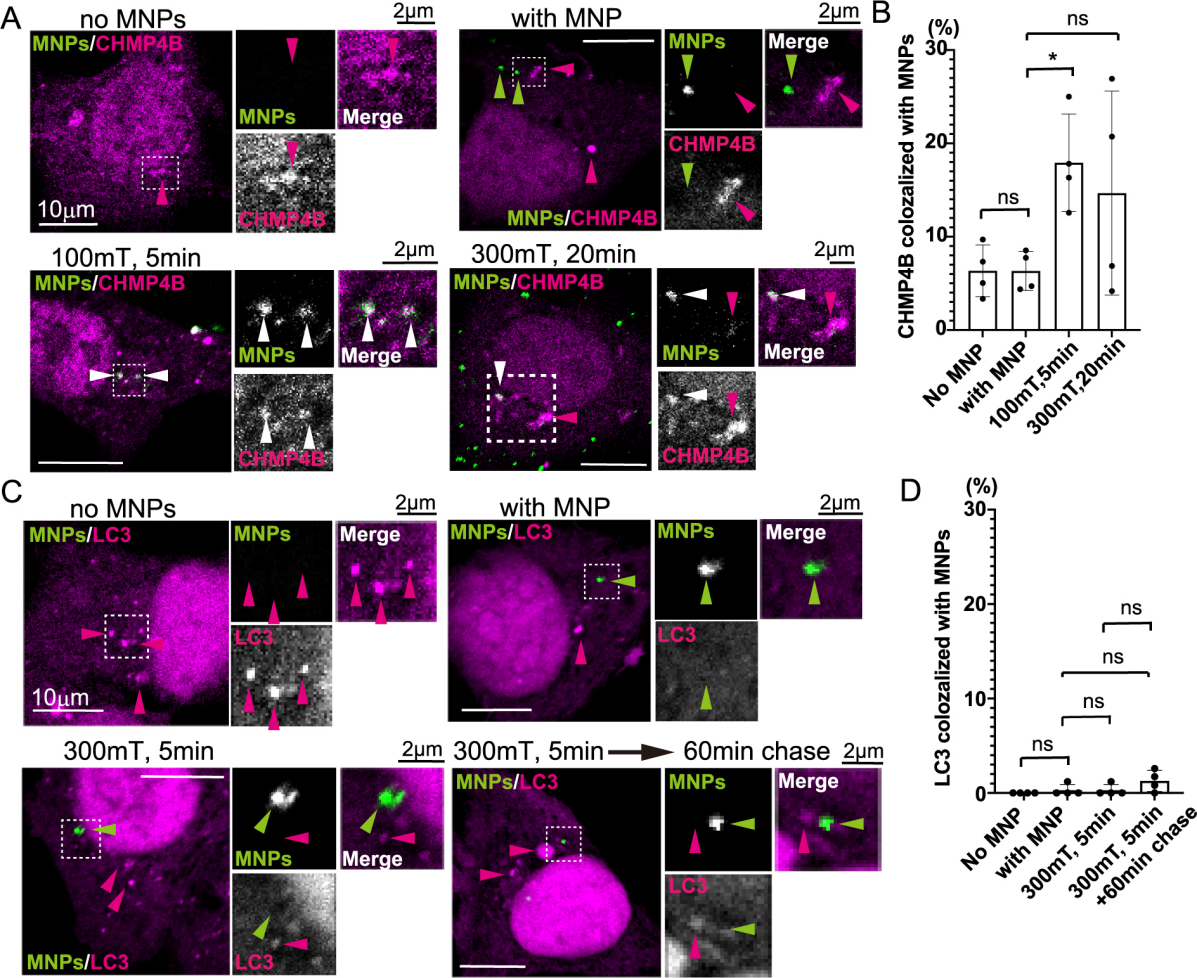
CHMP4B, but not LC3, was recruited to MNPs under a magnetic field (A) MCF7 cells transfected with CHMP4B-DsRed (magenta) were incubated with MNPs (green) for 40 min to allow internalization. After washing, a magnetic field was applied as indicated. The cells were fixed and observed using confocal microscopy. Scale bar; 10 μm. The dashed-line regions are enlarged in the insets, with their respective scale bars at 2 μm. Green arrowheads indicate MNPs without colocalization, magenta arrowheads indicate CHMP4B foci without colocalization with MNPs, and white arrowheads indicate CHMP4B foci colocalized with MNPs. Note that at 100 mT for 5 min, CHMP4B foci colocalized with MNPs. (B) The ratio of CHMP4B foci colocalized with MNPs to the total CHMP4B foci was calculated from the experiment in (A). More than 10 cells were analyzed, and the experiment was repeated four times. The bar graphs are presented as mean ± SD. A Mann–Whitney test was performed as indicated. *p<0.05; ns, not significant. (C) MCF7 cells transfected with DsRed-LC3 (magenta) were processed as described in (A). Dashed-line regions are enlarged. Magenta arrowheads indicate LC3 foci without colocalization with MNPs, and green arrowheads indicate MNPs without colocalization with LC3. (D) The ratio of LC3 foci colocalized with MNPs to the total LC3 foci was calculated from the experiment in (C). More than 10 cells were analyzed, and the experiment was repeated four times. The bar graphs are presented as mean ± SD. A Mann–Whitney test was performed as indicated. ns, not significant.

To investigate whether other molecules associated with membrane damage are recruited under a magnetic field applied via a neodymium magnet, we analyzed CHMP4B and LC3. MCF7 cells overexpressing CHMP4B-DsRed were internalized MNPs for 40 min, followed by application of magnetic fields of 100 mT for 5 min and 300 mT for 20 min using a neodymium magnet. Cells without MNPs were included as controls. Few CHMP4B foci were observed in the absence or presence of MNPs without magnetic field application. However, a significant increase in the colocalization of CHMP4B foci with MNPs was observed under a 100 mT magnetic field for 5 min (Fig. 5A and B, no MNPs: 6.4%; with MNPs: 6.3%; 100 mT, 5 min: 17.9%, *p*<0.05; 300 mT, 20 min: 14.7%, ns). Although colocalization increased under a 300 mT magnetic field for 20 min, the results often lacked reproducibility and were not statistically significant. These findings suggest that CHMP4B recruitment to MNPs occurs under a 100 mT magnetic field for 5 min.

Next, we analyzed LC3 recruitment. The MCF7 cells overexpressing DsRed-LC3 were subjected to a 300 mT magnetic field for 5 min. No significant recruitment of LC3 to MNPs was observed (Fig. 5C). While Gal3, CHMP4B and Eqt-SM are known to be recruited to damaged organelles within 10 min (Mercier *et al.*, 2020; Niekamp *et al.*, 2022; Thurston *et al.*, 2012), LC3 recruitment typically occurs after the recruitment of adaptor proteins (Fujita *et al.*, 2013; Wong and Holzbaur, 2014). Although LC3 can be recruited to damaged organelles as early as 5–6 min (Gomes-da-Silva *et al.*, 2019; Kageyama *et al.*, 2011), its localization in damaged organelles may persist for over an hr (Gomes-da-Silva *et al.*, 2019; Kageyama *et al.*, 2011; Maejima *et al.*, 2013). To test whether LC3 recruitment occurs over a longer time frame, we performed a 60-min chase period following magnetic field application. However, LC3 recruitment to MNPs remained undetectable (Fig. 5C). Quantification of LC3 foci colocalized with MNPs under a magnetic field revealed no significant differences compared to samples without a magnetic field (Fig. 5D, no MNP: 0%, with MNP: 0.3%, 300 mT, 5 min: 0.3%, 300 mT, ns, 5 min + 60 min chase: 1.3%, ns). In conclusion, CHMP4B, but not LC3, was recruited to damaged organelles by MNPs upon magnetic field application.

### Cell viability with magnetic field

**Fig. 6:**
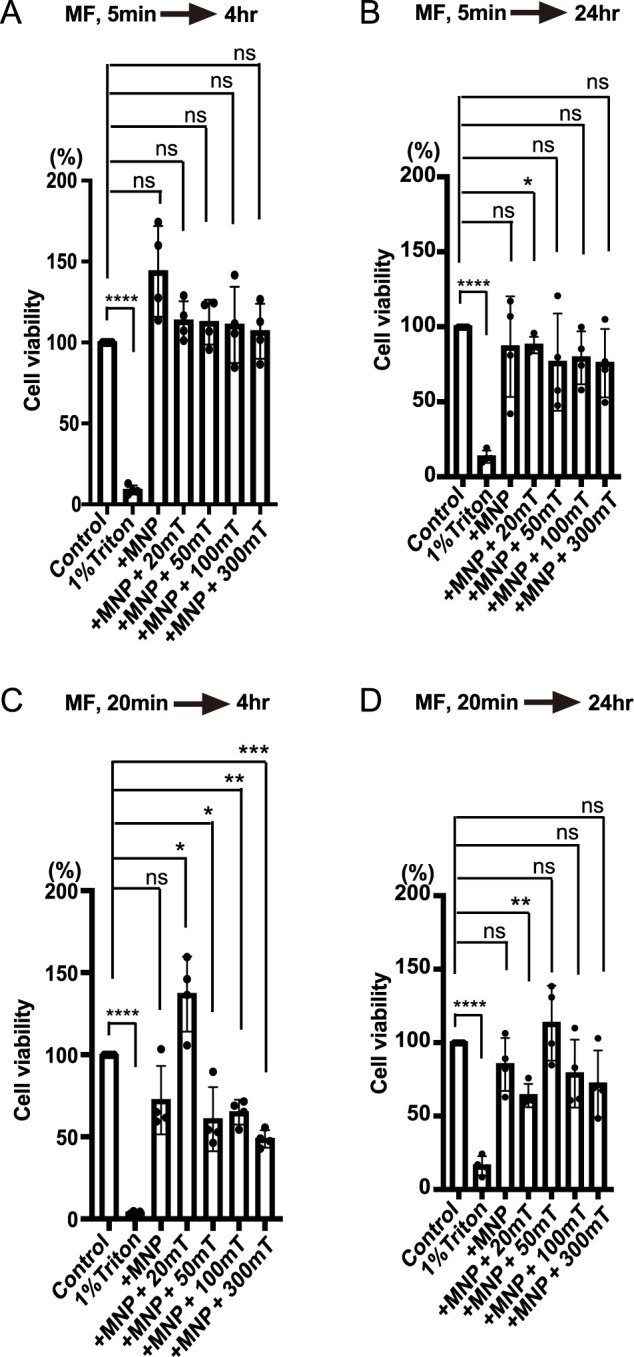
Cell viability under magnetic field exposure (A) MCF7 cells were incubated with 100 μg/ml MNPs for 20 min to allow internalization, followed by washing and the application of a magnetic field for 5 min as indicated. The cells were then incubated for 2 hr, after which MTT reagent was added and the cells were incubated for an additional 2 hr before lysis (total 4 hr post-magnetic field application). The experiment was repeated four times. Cell viability was calculated as a percentage relative to cells without MNPs, which were set as 100%. The bar graphs are presented as mean ± SD. A one-sample t-test and Wilcoxon test were performed to compare each sample to the 100% control. Cells treated with 1% Triton X-100 served as a toxicity control. ****p<0.0001, ns, not significant. (B) The experiment was performed as described in (A), but the cells were incubated for 22 hr after magnetic field application (total 24 hr post-magnetic field application) before processing for the MTT assay. A one-sample t-test and Wilcoxon test were performed. *p<0.05, ****p<0.0001, ns, not significant. (C) The experiment was performed as described in (A), but with magnetic field application for 20 min. Cells were incubated for 2 hr and processed for the MTT assay (total 4 hr post-magnetic field application). A one-sample t-test and Wilcoxon test were performed. *p<0.05, **p<0.01, ***p<0.001, ****p<0.0001, ns, not significant. (D) The experiment was performed as described in (C), but the cells were incubated for 22 hr after magnetic field application (total 24 hr post-magnetic field application). A one-sample t-test and Wilcoxon test were performed. **p<0.01, ****p<0.0001, ns, not significant.

We examined the potential cytotoxic effects of a magnetic field using the MTT assay. MCF7 cells were incubated with 100 μg/ml MNPs for 20 min, followed by the application of a magnetic field for either 5 or 20 min. After 2 or 22 hrs of incubation to allow for cell death processes, the MTT assay was conducted by adding the reagent to living cells for 2 hrs, lysing the cells, and measuring cell viability via MTT signal detection (Fig. 6). Cells incubated in medium alone served as the 100% viability control, while those treated with 1% Triton X-100 represented a high-toxicity control. Minimal toxicity was observed after 5 min of magnetic field exposure, regardless of 4 or 24 hrs of incubation (Fig. 6A and B). In contrast, 20 min of magnetic field exposure led to increased cytotoxicity after 4 hrs of incubation (Fig. 6C, Control: 100%, 1% Triton: 43.9%, +MNP: 71.5%, ns, 20 mT: 136.6%, *p*<0.05, 50 mT: 60.0%, *p*<0.05, 100 mT: 64.9%, *p*<0.01, 300 mT: 48.7%, *p*<0.001). However, after 24 hrs, cytotoxicity was observed only at 20 mT (Fig. 6D, Control: 100%, 1% Triton: 16.1%, +MNP: 85.1%, 20 mT: 63.9%, *p*<0.01, 50 mT: 113.3%, ns, 100 mT: 78.8%, ns, 300 mT: 71.9%, ns). These findings suggest that magnetic field exposure exceeding 50 mT for 20 min induces cytotoxicity, but this effect is mitigated after 24 hrs of recovery.

## Discussion

This study investigated the magnetic field intensity necessary to damage organelle membranes following the internalization of superparamagnetic MNPs. Endosomal membranes were found to be damaged at magnetic field strengths exceeding 20 mT of a DC magnetic field, with 100 mT identified as the most effective (Fig. 2). A 5-min magnetic field application was sufficient to induce damage, and extending the duration to 20 min did not increase the extent of membrane damage (Fig. 3). We confirmed that the damaged organelles were early endosomes, as Gal3 colocalized with EEA1/MNPs under a 100 mT magnetic field for 5 min, but not with Lamp1/MNPs (Fig. 4). CHMP4B recruitment was observed at 100 mT magnetic field for 5 min, whereas LC3 recruitment was not detected (Fig. 5). We speculate that damaged early endosomes could recover, as evidenced by the MTT assay results showing cytotoxicity after 4 hrs of a magnetic field exposure but not after 24 hrs (Fig. 6).

We observed a decrease in the colocalization of MNPs with Gal3 following exposure to 200 and 300 mT magnetic field for 20 min, but not at 100 mT for 20 min (Fig. 3F). This reduction in MNP colocalization was also observed with both Gal3 and CHMP4B (Fig. 4A, B and Fig. 5A and B). Several possible explanations may account for this phenomenon. The first possibility is that MNPs are transported to lysosomes via normal transport pathway during the 20-min magnetic field application. However, if this were the case, the application of a 100 mT magnetic field for 20 min should also reduce the colocalization of MNPs with Gal3, which was not observed (Fig. 3E and F). The second possibility is that MNPs are removed from damaged organelles via autophagy pathway. However, we did not observe LC3 recruitment following the application of a 300 mT magnetic field (Fig. 5C and D). The third possibility is that MNPs may penetrate the membrane of early endosomes and relocate to the cytosol under magnetic field conditions exceeding 200 mT. This hypothesis, however, requires further investigation, as discussed below.

Previously, it was reported that MNPs coated with ligands binding to membrane receptors could damage lysosomal membranes through alternating magnetic fields (AMF) or rotating magnetic fields (RMF) (Lopez *et al.*, 2022). Membrane damage in these studies was attributed to the mechanical shaking of membrane molecules bound to MNPs in a magnetic field (Golovin *et al.*, 2015). This mechanism suggests that little to no additional force is required for membrane disruption. Indeed, cationic latex beads or MNPs have been shown to damage membranes without applying external forces (Fujita *et al.*, 2013; Takahashi *et al.*, 2017), implying that the restriction of membrane molecule motion by binding to MNPs or latex beads alone is sufficient for disruption. In contrast, our study utilized MNPs without ligand modifications or cationic charges, which were observed to damage membranes mainly under a DC magnetic field exceeding 50 mT rather than an AC field (Fig. 2). Internalization of MNPs alone did not cause automatic membrane damage; instead, magnetic field application was essential for efficient membrane disruption (Fig. 4A, B, Fig. 5A and B). These results could imply that MNPs can damage membrane by physical force upon application of magnetic field. However, the bilayer membrane can be pulled out separately from adjoining membrane without rupture (Amador *et al.*, 2021). In our experiments, forces were applied from within the lumen, differing from external pulling forces. However, the forces produced by MNPs may be insufficient to cause significant membrane damage. Further analysis, including precise quantification of MNPs and the forces they generate, is required. In this study, we observed Gal3 recruitment to MNPs/EEA1 foci under a magnetic field, indicating that early endosome membranes were at least partially disrupted. Future work should focus on using electron microscopy to quantify the number of MNPs required to damage early endosomes and employing live-cell imaging to analyze MNP movement during magnetic field application.

Using an electromagnet, we observed a significant increase in the colocalization of MNPs with Gal3 at 20 mT for 5 min (Fig. 2A and E). However, no significant colocalization increase was detected at 20 mT when using a neodymium magnet (Fig. 3A and E). This discrepancy can be attributed to differences in the setups for the electromagnet and neodymium magnet. For the electromagnet, the magnetic field was applied outside the CO_2_ incubator by positioning the bottom of a 24-well plate against the electromagnet for 5 min (Supplementary Fig. 2A). In contrast, the magnetic field was applied inside a CO_2_ incubator, where a custom stage was prepared to position the neodymium magnet (Supplementary Fig. 2C and D). This setup provided better cell conditions with the neodymium magnet. Moreover, the direction of the magnetic field varied between the two systems: the electromagnet generated a horizontal field between the two poles of the yoke, whereas the neodymium magnet generated a vertical field. As the force exerted by MNPs depends on the magnetic field gradient, it is possible that the electromagnet produced a steeper gradient compared to the neodymium magnet.

In this study, we demonstrated that MNPs localized within the lumen of early endosomes can damage endosomal membranes under a static magnetic field. We were able to estimate the minimum force necessary to induce membrane damage from within the lumen. These findings contribute to a deeper understanding of the membrane properties of individual organelles and pave the way for developing novel techniques for precise MNP manipulation.

## Materials and Methods

### Reagents

The pCAG-human CHMP4B was kindly provided by Dr. Eiji Morita (Hirosaki University, Japan). and was inserted into the pcDNA3.1 vector containing a C-terminal DsRed tag using HindIII and EcoRI. The hGH-Eqt-SM-mKate2 plasmid, kindly provided by Dr. Christopher Burd (Yale University School of Medicine, USA), was modified to remove the signal sequence using the primers: 5'-GCGGGATCCATGTCCGCAGACGTGGCGGC-3' and 5'-AAGAATTCAGCTTTGCTCACGTGAATTTCA-3'. The amplified product was digested with BamHI and EcoRI and inserted into the pcDNA3.1 vector with a C-terminal DsRed tag. Additional constructs included: pEGFP-hLC3B provided by Eiki Kominami & Isei Tanida (Addgene plasmid #87872) (Tanida *et al.*, 2001), was inserted into pcDNA3.1 with an N-terminal DsRed tag using *BamH*I and *Xho*I. pmCherry-hGal3, provided by Hemmo Meyer (Addgene plasmid #85662) (Papadopoulos *et al.*, 2017). hLAMP1-mScarletI, provided by Dorus Gadella (Addgene plasmid #98827) (Chertkova *et al.*, 2020). Antibodies used include mouse monoclonal anti-EEA1 (3C10; MBL, Tokyo, Japan) and anti-Lamp1 (H4A3; Abcam, Cambridge, UK). Secondary antibodies conjugated to Alexa Fluor 594, Alexa Plus 488, or Dylight 405 were obtained from Thermo Fisher Scientific (Waltham, MA, USA). DAPI was purchased from Merck (Darmstadt, Germany).

### Cell culture

MCF7 human mammary carcinoma cells were obtained from RIKEN BRC (Tsukuba, Japan) and cultured in DMEM (Nacalai Tesque, Kyoto, Japan) supplemented with: 10% FBS (Regular; CORNING, NY, USA), 1x MEM non-essential amino acids solution (Wako, Osaka, Japan), and 1 mmol/L sodium pyruvate solution (Wako). Cells were maintained at 37°C in a 5% CO_2_ incubator.

### MNPs

MNPs were synthesized as described previously (Kawamura *et al.*, 2024). Briefly, 15 nm Fe_3_O_4_ MNPs were synthesized via a thermal decomposition method and stabilized with hyperbranched polyglycidol for water solubility. The MNPs were labeled with fluorescein isothiocyanate (FITC) for fluorescence detection, resulting in 20 nm hbPGYLD-Fe_3_O_4_-FITC particles. Previously, we tested the cytotoxicity of hbPGYLD-Fe_3_O_4_-FITC with MCF7 cells, and found more than 80% of the cells were viable when treated with 100–200 μg/ml of MNPs for 48 h (Kawamura *et al.*, 2024). Therefore, we used 100 μg/ml of MNPs usually. As MNP signal became very weak after permeabilization, we used 400 μg/ml of MNPs in Fig. 4.

### Immunofluorescence

MCF7 (7 × 10^4^) cells were seeded on coverslips and transfected with plasmids using Lipofectamine LTX (Thermo Fisher Scientific) following the manufacturer’s protocol. After 16–24 hrs of transfection, the cells were treated with 100 μg/ml MNPs for designated times, fixed with 4% paraformaldehyde for 15 min, quenched by 50 mM NH_4_Cl/PBS, and stained with DAPI. Secondary antibodies were applied as required, and confocal images were captured using Nikon C2 and AX/AXR microscopes with 60× objectives (NA1.40 and NA1.42).

For LLOMe treatment, 1 mM LLOMe (Cayman Chemical, MI, USA) was treated for 10 min, followed by washing and fixation. After permeabilization and blocking with 0.02% saponin/5% BSA/PBS for 30 min, the cells were incubated with anti-EEA1 or anti-Lamp1 antibodies in 0.02% saponin/PBS for 2 hrs. Secondary staining was performed using goat anti-mouse IgG conjugated with Alexa Fluor 594 in PBS for 45 min. The cells were then mounted with Mowiol.

In Fig. 4, after fixation, the cells were permeabilized with 50 μg/ml digitonin (Tokyo Chemical Industry, Tokyo, Japan) for 5 min and quenched with 50 mM NH4Cl/PBS for 20 min. The cells were then blocked with 5% BSA/PBS for 30 min and stained with primary antibodies in PBS overnight, and processed with secondary antibodies conjugated to Dylight 405.

### Image analysis and statistics

For quantification, identical settings were used to capture images from the confocal microscope for both controls and experimental samples. Areas containing red fluorescence and MNPs were randomly selected for analysis. ImageJ Fiji software was employed for image analysis (Schindelin *et al.*, 2012). For colocalization experiments, the number of foci per cell was counted, and the percentage of foci with colocalized red fluorescence was calculated relative to the total number of foci in each experiment. Images were captured from more than ten cells per experiment, and each experiment was repeated four times. Each point in the bar graphs represents the ratio of colocalized MNPs calculated from over ten cells per experiment. Typically, more than 50 MNPs were analyzed in each experiment. Statistical analyses were performed using GraphPad PRISM version 10 (GraphPad Software, San Diego, California, USA; www.graphpad.com).

### Magnetic field application using an electromagnet

The electromagnet was constructed with a magnetic yoke consisting of two magnets connected by 200 windings of wire. The electromagnet was connected to a Bipolar Amplifier (Takasago, Kanagawa, Japan) and Multifunction Generator (WF1973; NF CORPORATION, Kanagawa, Japan) to supply electricity. The magnetic yoke was positioned directly beneath the bottom of a 24-well plate containing MCF7 cells (Supplementary Fig. 2A). A magnetic field was applied for 5 min using the electrical setup (Supplementary Fig. 2B). The resulting magnetic field strength within the 24-well plate was approximately 20 mT, as measured by a Gauss meter (MG-801; MAGNA, Tokyo, Japan).

### Magnetic field application by neodymium magnet

A neodymium magnet (NTS2069, AS ONE, Osaka, Japan) was used to generate a DC magnetic field. Five custom stages were fabricated using a 3D printer (Raise3D Pro2) with ABS material at the Advanced Prototyping Processing Center, Iwate University (Supplementary Fig. 2D). These stages were designed to produce magnetic field strengths of 20, 50, 100, 200, and 300 mT. The height of each stage was calibrated by measuring the magnetic field using a Gauss meter (Supplementary Fig. 2C). The neodymium magnet was positioned on the respective stage to apply the desired magnetic field, and cells were incubated on the stage at 37°C in a 5% CO_2_ incubator as specified in the text.

### MTT assay

MTT assay was performed as previously described (Kawamura *et al.*, 2024). Briefly, 3 × 10^4^ MCF7 cells were seeded to 96 well-plates. The cells were incubated with 100 μg/ml MNPs for 20 min, followed by washing and application of a magnetic field as indicated. After 2 or 22 hrs of incubation, MTT reagent (345-01821, FUJIFILM) was added to the cells (final concentration: 5 mg/ml) and incubated for 2 hrs at 37°C. The MTT signal was measured as described previously (Kawamura *et al.*, 2024). As controls, cells were incubated either with medium alone or with medium containing 1% Triton X-100, and processed as described above. The signal from cells treated with medium alone was set as 100% in each experiment, and cell viability was calculated as a percentage of this control. Statistical analysis was performed using one-sample t-tests and Wilcoxon tests.

## Disclaimer

This study involves genetic modification techniques, which were conducted in compliance with all relevant ethical guidelines. The authors declare no conflicts of interest related to this research.
