# Supplementary material for: Magnetic control of membrane damage in early endosomes using internalized magnetic nanoparticles
Source: Cell Struct Funct. 2024 Dec 27;50(1):25–39. doi: 10.1247/csf.24037 (PMC12702682; doi:10.1247/csf.24037)
Supplement: Supplementary file 1 — Supplementary Materials [file csf_50_24037_1.zip › 50_24037_Supplementary_Fig_Legend.docx]

**Supplementary Figure 1. Gal3 colocalization with Lamp1 following LLOMe treatment. A.** MCF7 cells overexpressing mCherry-Gal3 (magenta) were treated with either DMSO or 1 mM LLOMe for 10 min, then fixed and stained with anti-EEA1 antibody (green) and DAPI (not shown). Magenta arrowheads indicate Gal3 foci, and white arrowheads indicate Gal3 foci colocalized with EEA1. Dashed-line regions are enlarged in the insets. Scale bar: 10 µm. **B.** Similar experiments were performed as described in (A), but cells were stained with anti-Lamp1 antibody (green). White arrowheads indicate Gal3 foci colocalized with Lamp1, while magenta arrowheads indicate Gal3 foci not colocalized with Lamp1. **C.** The number of Gal3 foci colocalized with EEA1 in (A) was quantified and presented for each experiment. More than 10 cells were analyzed per experiment, and the experiment was repeated six times. The bar graphs are presented as mean ± SD. A Mann–Whitney test was performed. ns, not significant. **D.** The number of Gal3 foci colocalized with Lamp1 in (B) was quantified as in (C). A Mann–Whitney test was performed. ***p* < 0.01.

**Supplementary Figure 2. Magnetic field setup and application. A.** Photograph of the magnetic field setup using an electromagnet. The electromagnet was positioned beneath the bottom of a 24-well plate. **B.** Patterns of DC and AC magnetic fields. A 2 V DC magnetic field and a 6 V AC magnetic field at 0.5 Hz were applied. **C.** Magnetic field setup using a neodymium magnet. The height between the neodymium magnet and the 24-well plate was adjusted to achieve the desired magnetic field intensity. **D.** Photograph of the stage fabricated using a 3D printer. The stage was placed beneath the 24-well plate, with the neodymium magnet positioned inside the stage.

**Supplementary Figure 3. Magnetic field application alone did not damage early endosome membranes. A.** MCF7 cells transfected with mCherry-Gal3 (red) were subjected to a magnetic field as indicated. The cells were fixed and stained with anti-EEA1 antibody (green). Magenta arrowheads indicate Gal3 foci that do not colocalize with EEA1. Scale bar; 10µm. The dashed-line regions are enlarged in the insets, with their respective scale bars at 2 µm. **B.** The number of Gal3 foci colocalized with EEA1 per cell is shown from the experiment described in (A). More than 10 cells were analyzed, and the experiment was repeated four times. The bar graphs are presented as mean ± SD. For comparison, data from Fig. 4B were included for 300 mT for 20 min with MNPs. A Mann–Whitney test was performed. **p* < 0.05; ns, not significant.
