# Supplementary material for: Magnetic control of membrane damage in early endosomes using internalized magnetic nanoparticles
Source: Cell Struct Funct. 2024 Dec 27;50(1):25–39. doi: 10.1247/csf.24037 (PMC12702682; doi:10.1247/csf.24037)
Supplement: Supplementary file 1 — Supplementary Materials [file csf_50_24037_1.zip › 50_24037_Supplementary_Fig_3.pdf]

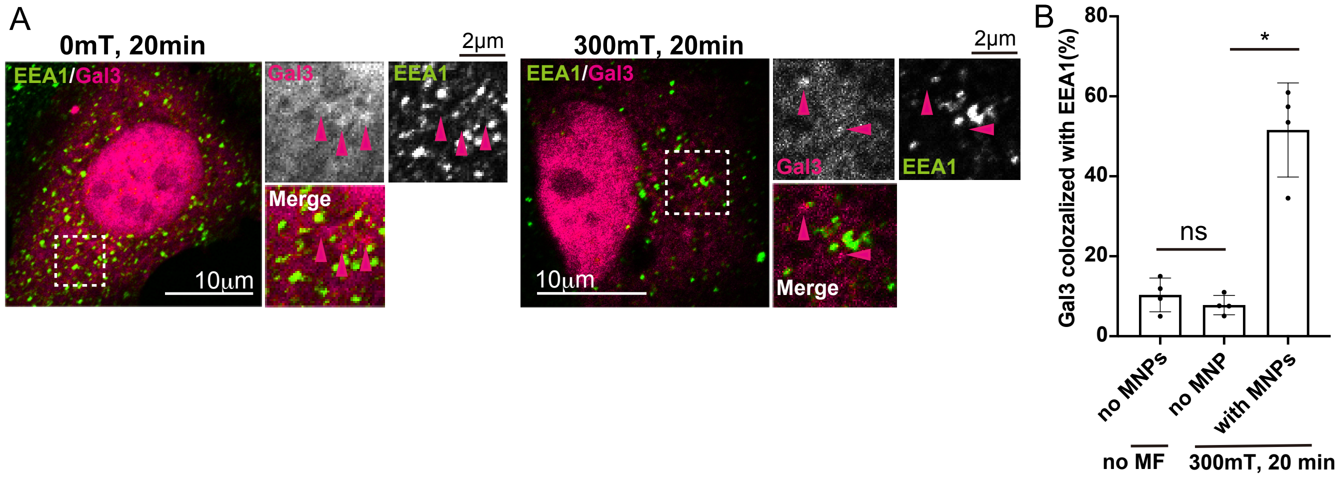

Supplementary Figure 3. Magnetic field application alone did not damage the early endosome membranes.
