# Supplementary figures and images for: Magnetic control of membrane damage in early endosomes using internalized magnetic nanoparticles
Source: Cell Struct Funct. 2024 Dec 27;50(1):25–39. doi: 10.1247/csf.24037 (PMC12702682; doi:10.1247/csf.24037)

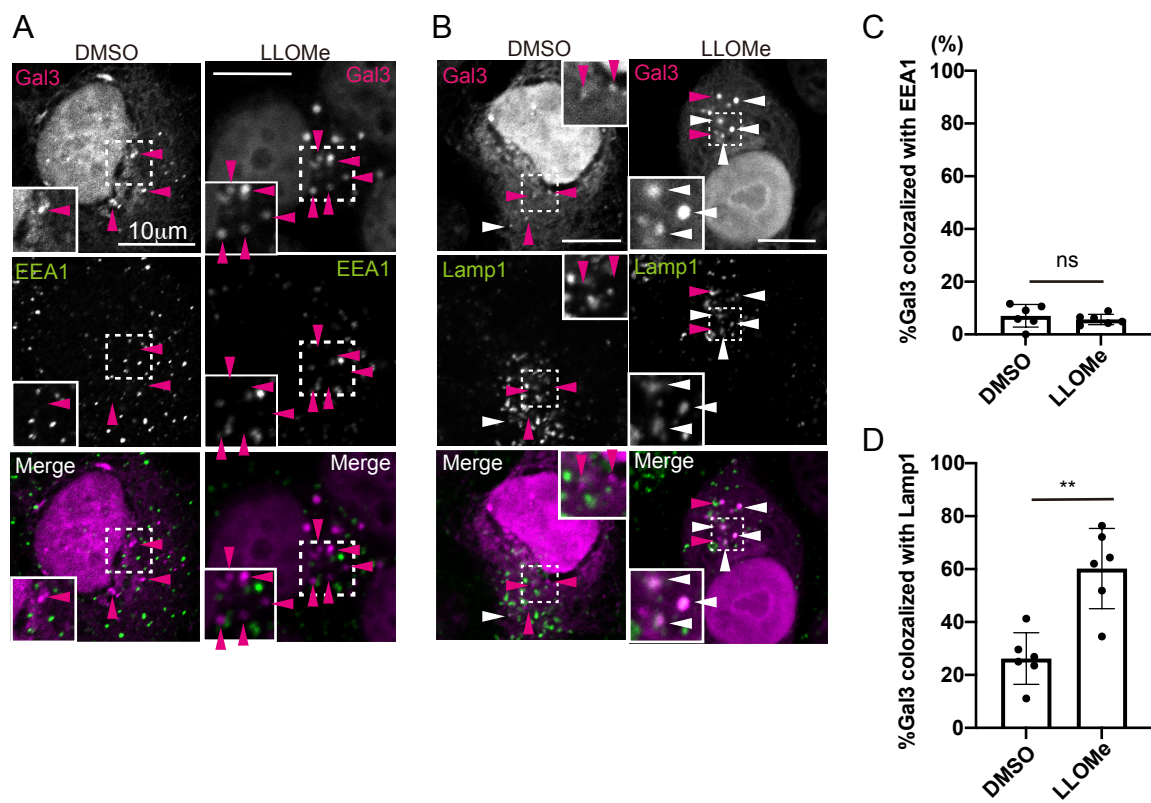

Supplementary Figure 1. Gal3 colocalization with Lamp1 following LLOMe treatment.

Supplement: Supplementary file 1 — Supplementary Materials [file csf_50_24037_1.zip › 50_24037_Supplementary_Fig_1.pdf]

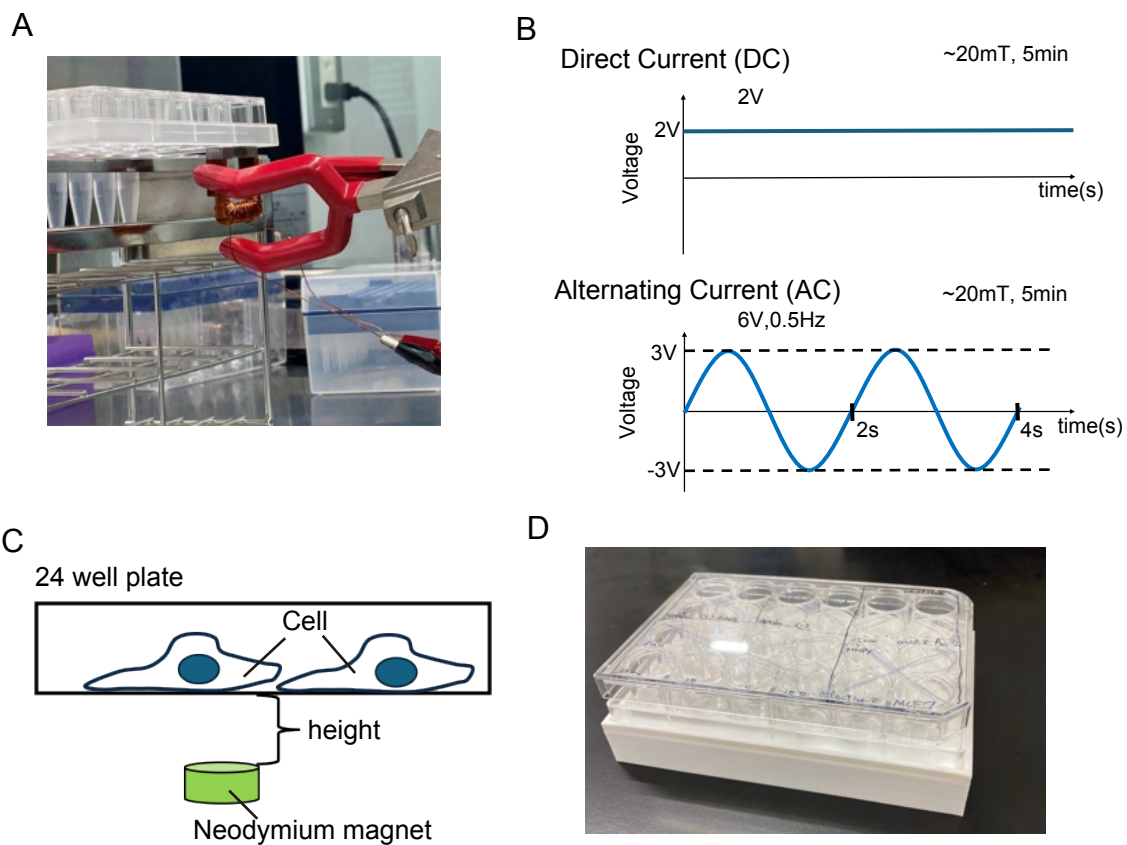

**Supplementary Figure 2. Magnetic field setup and application.**

Supplement: Supplementary file 1 — Supplementary Materials [file csf_50_24037_1.zip › 50_24037_Supplementary_Fig_2.pdf]
